# Predicting Escalation of Care for Childhood Pneumonia Using Machine Learning: Retrospective Analysis and Model Development

**DOI:** 10.2196/57719

**Published:** 2025-03-04

**Authors:** Oguzhan Serin, Izzet Turkalp Akbasli, Sena Bocutcu Cetin, Busra Koseoglu, Ahmet Fatih Deveci, Muhsin Zahid Ugur, Yasemin Ozsurekci

**Affiliations:** 1 Department of Pediatrics, Hacettepe University Medical School, Gevher Nesibe Avenue, Altindag, Ankara, 06230, Turkey, 90 3051350; 2 Department of Health Information Systems, University of Health Sciences, Istanbul, Turkey; 3 Department of Pediatric Infectious Diseases, Hacettepe University Medical School, Ankara, Turkey

**Keywords:** childhood pneumonia, community-acquired pneumonia, machine learning, clinical decision support system, prognostic care decision

## Abstract

**Background:**

Pneumonia is a leading cause of mortality in children aged <5 years. While machine learning (ML) has been applied to pneumonia diagnostics, few studies have focused on predicting the need for escalation of care in pediatric cases. This study aims to develop an ML-based clinical decision support tool for predicting the need for escalation of care in community-acquired pneumonia cases.

**Objective:**

The primary objective was to develop a robust predictive tool to help primary care physicians determine where and how a case should be managed.

**Methods:**

Data from 437 children with community-acquired pneumonia, collected before the COVID-19 pandemic, were retrospectively analyzed. Pediatricians encoded key clinical features from unstructured medical records based on Integrated Management of Childhood Illness guidelines. After preprocessing with Synthetic Minority Oversampling Technique–Tomek to handle imbalanced data, feature selection was performed using Shapley additive explanations values. The model was optimized through hyperparameter tuning and ensembling. The primary outcome was the level of care severity, defined as the need for referral to a tertiary care unit for intensive care or respiratory support.

**Results:**

A total of 437 cases were analyzed, and the optimized models predicted the need for transfer to a higher level of care with an accuracy of 77% to 88%, achieving an area under the receiver operator characteristic curve of 0.88 and an area under the precision-recall curve of 0.96. Shapley additive explanations value analysis identified hypoxia, respiratory distress, age, weight-for-age *z* score, and complaint duration as the most important clinical predictors independent of laboratory diagnostics.

**Conclusions:**

This study demonstrates the feasibility of applying ML techniques to create a prognostic care decision tool for childhood pneumonia. It provides early identification of cases requiring escalation of care by combining foundational clinical skills with data science methods.

## Introduction

Pneumonia is responsible for 14% of all mortality in children aged <5 years and is included in World Health Organization (WHO) reports as the cause of death in 740,180 children in 2019 alone [1,2]. The Global Action Plan for the Prevention and Control of Pneumonia and Diarrhea, which was released by the WHO and UNICEF, aimed to reduce the mortality rate from pneumonia and diarrhea in children aged <5 years [2,3]. They have set targets that include vaccination, water and air sanitation, exclusively breastfeeding in the first 6 months, and eliminating pediatric HIV cases, along with appropriate pneumonia and diarrhea care.

It has been demonstrated that timely and accurate diagnosis of pneumonia and appropriately initiated treatment reduce mortality by up to 28% [4]. Diagnosis can often be difficult, since the clinical presentation of pneumonia in children is variable [5]. For this reason, the WHO has published the Integrated Management of Childhood Illness (IMCI) guidelines, which guide physicians in diagnosing, treating, and identifying danger signs of pneumonia [6]. While some cases of pneumonia are treatable with appropriate interventions, even low-cost or low-tech options [1], pneumonia remains a leading cause of morbidity and mortality, particularly in resource-limited countries and regions [2]. Managing high-risk populations continues to present significant challenges, especially in intensive care settings where patients often require advanced respiratory support. In addition, it has been shown that families seeking health services in resource-limited settings causes delays in providing appropriate treatment, leading to disease progression [7]. These highlight the need to improve medical care decisions, particularly in regions with limited resources, to reduce pneumonia-related morbidity and mortality.

Early and accurate recognition of patients who may require escalation of care to tertiary facilities is essential, particularly for those who will require mechanical ventilation or advanced respiratory support [8]. Predicting which patients will deteriorate is challenging due to the heterogeneous presentation of pneumonia, and clinical features such as hypoxia, respiratory distress, nutritional status, and comorbidities are critical markers that necessitate closer monitoring or transfer [9,10]. Prolonged duration of illness and failure to respond to initial treatments are also important as they may indicate inadequate treatment, misdiagnosis, or incorrect identification of potential pathogens, which can lead to the escalation of care [7,11].

Data science can provide actionable evidence for effective clinical intervention in pediatric diseases in the future [12] and can reduce inequality in health care [13]. Also, using big data and machine learning (ML) technologies is promising for childhood pneumonia in low- and middle-income countries (LMICs), especially patient-risk stratification for developing severe disease and mortality [14]. Because of their flexibility and high accuracy, ML models are used in medicine in the fields of prediction (prognostics) and classification (diagnostics) [12]. Additionally, the use of ML offers great promise for decision support in managing community-acquired pneumonia (CAP) in children, as demonstrated in recent studies. These include predicting intensive care unit needs [15], low-cost and noninvasive diagnostics for childhood pneumonia in resource-limited settings [16], supporting pathogen identification at admission only using basic clinical and laboratory features [11], and using natural language processing with ML for supporting clinical decisions on radiology reports [17].

It has been seen that the vast majority of data science studies on pneumonia aims to provide diagnostic support to the physician by processing radiological images [18]. However, diagnostic utilities are mostly unavailable in LMICs and primary care units. Therefore, physicians need prognostic support algorithms that distinguish between serious and nonserious cases without using advanced diagnostic equipment.

We aimed to develop an ML-based clinical decision support tool for childhood pneumonia that can be used by non–intensive care physicians, particularly those working in LMICs, in predicting the escalation of care and thereby ensuring the effective diagnosis and treatment of pneumonia, which is one of the 2025 goals of the WHO [1,3].

## Methods

### Case Definition and Patient Selection

Our study included pediatric patients who received inpatient treatment at Hacettepe University Medical School, a large, urban, tertiary, academic medical center in Ankara, Türkiye, between January 2014 and April 2020. The center serves a diverse range of pediatric patients from both urban and rural areas across the country, including those requiring advanced multidisciplinary care as well as those with less severe conditions. All patients were diagnosed with CAP based on the most recent IMCI guidelines, which provide a structured clinical framework focused on clinical features rather than advanced imaging or laboratory results [6,19]. Patients younger than 28 days of age (neonatal age), those older than 18 years, and those who had been hospitalized within the last 14 days were excluded.

The medical records of 437 patients were retrospectively examined by pediatricians, who encoded the candidate features from unstructured admission notes based on the IMCI guidelines (Tables1  2). These variables were chosen based on their clinical value in clinical decision-making and their availability in primary care.

**Table 1.** Candidate features: clinical variables.

Clinical variables	Description
Age	Age in months at the time of admission
Weight (*z* score)	Standardized score based on Turkish children reference values [20], indirectly reflecting nutritional status
Gender	Biological sex (male or female)
Complaint period	Duration (days) from symptom onset to admission
Comorbidity	Presence of any significant underlying medical conditions, including congenital disorders, genetic syndromes, neuromuscular diseases, and chronic respiratory or cardiac issues
Recent antibiotics usage	Prescribed oral antibiotic use within the 14 days before admission, suggesting an inadequately treated infection or failure to respond initial care
Fever	Presence of elevated body temperature at admission
Cough	A key respiratory symptom at admission
Loss of appetite	Sign of systemic illness, reflecting impact on the patient’s well-being
Respiratory distress	Presence of shortness of breath, rapid breathing (tachypnea), nasal flaring, or chest wall retractions at initial examination
Abnormal lung sounds	Auscultatory findings (eg, crackles or wheezing), indicative of pulmonary pathology at initial examination
Hypoxia	SaO_2_^a^ measured by pulse oximetry; hypoxia is defined as SaO_2_ below 92% at initial examination
Level of care severity	Primary outcome; whether the patient requires pneumonia care at a tertiary care unit, including PICU^b^ admission or respiratory support (oxygenation or ventilation), at any point during the hospital stay

a SaO_2_: peripheral blood oxygen saturation.

b PICU: pediatric intensive care unit.

**Table 2.** Candidate features: laboratory variables.

Laboratory variables	Unit
Hemoglobin	Grams per deciliter (g/dL)
Leukocytes	Cells per liter (×10^6^/L)
Lymphocytes	Cells per liter (×10^6^/L)
Neutrophils	Cells per liter (×10^6^/L)
Platelets	Cells per liter (×10^9^/L)
C-reactive protein	Milligrams per liter (mg/L)
Albumin	Grams per deciliter (g/dL)
Sodium	Milliequivalents per liter (mEq/L)
Aspartate aminotransferase	Units per liter (U/L)
Alanine aminotransferase	Units per liter (U/L)

The primary outcome was the “level of care severity,” scaled as severe or nonsevere. This categorization was made by physician-encoders based on whether the patient required referral to a tertiary care unit, using medical notes during the hospital stay. Children classified as severe included those admitted to the pediatric intensive care unit or those who required oxygenation or ventilation support at any time during the hospital stay.

### Ethical Considerations

This study’s design and procedures were approved by the Hacettepe University Clinical Research Ethics Committee with protocol GO-20/1182. Since this study is a retrospective analysis using previously collected data, informed consent was not required as per the ethics committee’s approval. All data used in this study were deidentified before analysis to ensure participant privacy and confidentiality. No compensation was provided to participants, as this study did not involve direct human participant recruitment.

### Study Population

This study included 437 hospitalized patients with CAP, categorized into nonsevere (n=133, 30.4%) and severe cases (n=304, 69.6%). Demographic and clinical candidate variables, along with laboratory indices, were collected. Group comparisons were made using the Mann-Whitney *U* test for continuous variables and the *χ*^2^ test for categorical variables, with significance set at *P*<.05. A summary of these characteristics and statistical comparisons are provided in Table 3.

**Table 3.** Characteristics of the study population by level of care severity (N=437).

Candidate variables	Nonsevere (n=133, 30.4%)	Severe (n=304, 69.6%)	Test statistic (*df*)	*P* value
Age (months), median (IQR)	44 (13 to 98)	23 (7 to 64.5)	16,602^a^	.003
Weight (*z* scores), median (IQR)	−0.57 (−1.4 to 0.45)	−0.7 (−2.5 to 0.4)	17,784^a^	.045
Complaint period (days), median (IQR)	4 (2 to 7)	4 (2 to 7)	19,274^a^	.44
Gender, n (%)			0.05^a^	.83
Male	68 (30.9)	152 (69.1)		
Female	65 (30)	152 (70)		
Comorbidity, n (%)	85 (28.7)	211 (71.3)	1.28^b^ (1)	.26
Recent antibiotic usage, n (%)	40 (26.3)	112 (73.7)	1.87^b^ (1)	.17
Fever, n (%)	100 (32.3)	210 (67.7)	1.68^b^ (1)	.20
Cough, n (%)	115 (31.3)	253 (68.8)	0.50^b^ (1)	.48
Loss of appetite, n (%)	37 (32)	80 (68)	0.11^b^ (1)	.74
Respiratory distress, n (%)	43 (17.1)	208 (82.9)	49.30^b^ (1)	<.001
Abnormal lung sounds, n (%)	102 (26.9)	277 (73.1)	16.70^b^ (1)	<.001
Hypoxia, n (%)	20 (7.7)	240 (92.3)	156.82^b^ (1)	<.001
Hemoglobin (g/dL), median (IQR)	11.6 (10.4 to 12.9)	11.6 (10.6 to 12.6)	20,022^a^	.87
Leukocytes (×10^6^/L), median (IQR)	9900 (6800 to 14,600)	10,950 (8050 to 15,850)	17,837^a^	.05
Lymphocytes (×10^6^/L), median (IQR)	2300 (1400 to 3700)	2800 (1900 to 4400)	17,039^a^	.01
Neutrophils (×10^6^/L), median (IQR)	5285 (2700 to 9200)	6500 (3650 to 10,900)	17,645^a^	.045
Platelets (×10^9^/L), median (IQR)	310 (225 to 386)	317.5 (230.5 to 425)	19,399^a^	.50
C-reactive protein (mg/L), median (IQR)	2.06 (0.79 to 7.67)	2.06 (0.83 to 7.35)	19,842^a^	.76
Albumin (g/dL), median (IQR)	3.9 (3.73 to 4.2)	3.9 (3.4 to 4.2)	17,121^a^	.01
Sodium (mEq/L), median (IQR)	136 (135 to 138)	136 (134 to 138)	19,657^a^	.64
Aspartate aminotransferase (U/L), median (IQR)	35 (26 to 42)	35 (28 to 50)	18,382^a^	.13
Alanine aminotransferase (U/L), median (IQR)	17 (12 to 26)	18 (13 to 29)	18,457^a^	.15

a Mann-Whitney *U* test.

b Chi-square test.

### Data Preprocessing

Data preprocessing, analysis, visualization, and model setup were conducted using Python (version 3.12; Python Software Foundation). We used Python libraries such as *Pandas*, *NumPy*, *Matplotlib*, *Seaborn*, and *Plotly* for exploratory data analysis. For model development, the *PyCaret* library was used, which includes an unsupervised anomaly detection module to identify and handle anomalous data points. *PyCaret* also offers various preprocessing modules to iteratively handle missing data using the light gradient boosting machine (LightGBM) algorithm. In this method, missing values were treated as dependent variables and predicted based on other available features, minimizing bias. Individual feature weights were applied during this process. Specifically, of the 415 cases, the following features had missing values: C-reactive protein (n=34, 8.2%), albumin (n=10, 2.4%), sodium (n=8, 1.9%), aspartate aminotransferase (n=16, 3.9%), and alanine aminotransferase (n=16, 3.9%). For numerical data, min-max scaling was applied, while categorical data were processed using one-hot encoding. These preprocessing steps ensured the dataset was well prepared for model training and validation.

### Handling the Imbalanced Dataset

The balance of the dataset was assessed using Shannon entropy, yielding a value of 0.7, which indicates an imbalanced dataset. To address this, we applied Synthetic Minority Oversampling Technique (SMOTE)–Tomek, a refined variation of the widely recognized SMOTE. This approach combines oversampling of the minority class with the removal of overlapping samples from the majority class through Tomek links. So, the ratio of samples becomes 1:1. The *Imblearn* library was used for implementing data oversampling.

The dataset was split into two sets using the *train_test_split* method of the *SciKit-Learn* library. In the beginning, we allocated 5% of the general dataset as test data in order to prevent data leakage. The remaining 95% was split into training (352/415, 85%) and validation (63/415, 15%) sets.

### Algorithms

*PyCaret* provides efficient implementations of state-of-the-art algorithms and is reusable among scientific disciplines. We used the *PyCaret* classifier module for classification, which includes the following models: ridge classifier, linear discriminant analysis, naïve Bayes, extra tree classifier, extreme gradient boosting (XGBoost), random forest, gradient boosting classifier, LightGBM, CatBoost classifier, logistic regression, k-neighbors classifier, decision tree, AdaBoost classifier, quadratic discriminant analysis, support vector machine with linear kernel, and dummy classifier.

**Figure 1.**
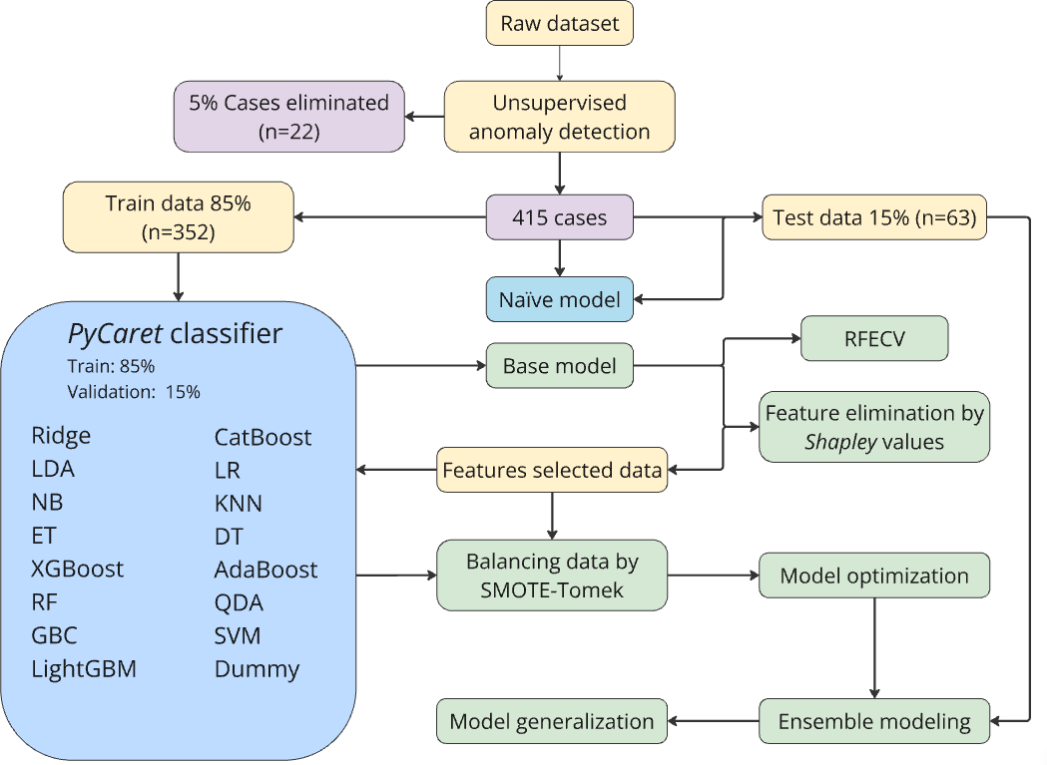
The experimental setup: in this figure, we illustrate the experimental process of our models. Initially, we cleaned the data by identifying 5% of cases as abnormal data using unsupervised learning. We then split the data into a train set (85%) and a validation set (15%) using the *PyCaret* classifier model. The base model with the highest AUC-ROC value was the RF algorithm. Subsequently, we determined the optimal number of features as 18 using RFECV and selected the top 18 features based on Shapley values. We then balanced the dataset using the SMOTE-Tomek method and developed high-performing models. After optimizing the hyperparameters, we selected the best-performing model and created new models by using ensemble methods. In parallel, we developed a new model using only clinical findings for clinical prediction. AdaBoost: AdaBoost classifier; AUC-ROC: area under the receiver operator characteristic curve; CatBoost: CatBoost classifier; DT: decision tree; Dummy: dummy classifier; ET: extra tree classifier; GBC: gradient boosting classifier; KNN: k-neighbors classifier; LDA: linear discriminant analysis; LightGBM: light gradient boosting machine; LR: logistic regression; NB: naïve Bayes; QDA: quadratic discriminant analysis; RF: random forest; RFECV: recursive feature elimination with cross-validation; Ridge: ridge classifier; SMOTE: Synthetic Minority Oversampling Technique; SVM: support vector machine linear kernel classifier; XGBoost: extreme gradient boosting.

In our work, we considered 10-fold cross-validation. While developing our model with *PyCaret* tools, we implemented the tuning function using the *Tune-Sklearn* library and the *hyper-band* optimization algorithm to obtain a set of best-performing parameters. For ensembling, we also used *PyCaret* classifier ensemble, stack, and blender methods. Ensembling methods have strong evidence that they can significantly enhance the accuracy of classifications [21]. After the optimization of parameters, in the last phase, we used the most common ensemble methods provided by the *PyCaret* library to further improve our model’s performance (Figure 1).

### Feature Selection and Data-Reducing Methods

**Figure 2.**
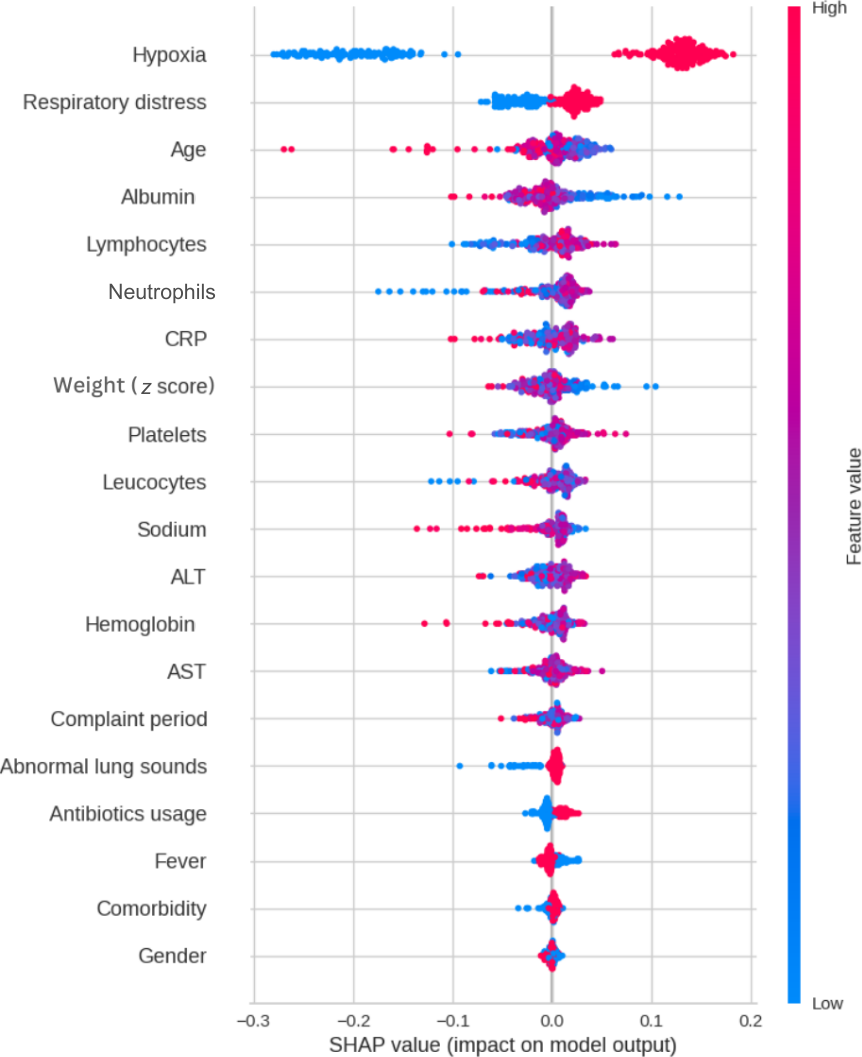
Feature selection: SHAP values are presented for the random forest classifier model with the highest AUC-ROC score in the dataset before feature selection, using the *SHAP* library’s *plot_summary* module. The y-axis shows the importance of each feature, with the most important feature at the top and the least important at the bottom. The colors represent the contribution of each feature to the model’s prediction. For example, features that have a large positive contribution to the prediction are shown in a warm color (eg, red), while features that have a large negative contribution are shown in a cool color (eg, blue). In this example, hypoxia is the most important attribute in the plot. The presence of hypoxia (hypoxia=1) causes the model to move closer to the target class, while its absence causes the model to move away from the target class. This predicts that hypoxia is an aggravating factor, while high levels of albumin have a protective effect for the target class. In summary, hypoxia is an adverse factor, and high albumin levels are protective. ALT: alanine aminotransferase; AST: aspartate aminotransferase; AUC-ROC: area under the receiver operator characteristic curve; CRP: C-reactive protein; SHAP: Shapley additive explanations.

Feature selection is a process of one-by-one evaluation to determine which features are effective on the results within the dataset. Irrelevant or partially relevant features can negatively impact ML model performance and make the ML model learn based on irrelevant features. These methods are aimed at eliminating irrelevant features and keeping the strong features to reduce the dimension of the dataset. Recursive feature elimination is a feature selection method that fits a model and removes the irrelevant features until the specified number of features is reached. Recursive feature elimination with cross-validation (RFECV) aims to select the optimal number of features using permutation importance and recursive feature elimination. In this study, we used the *RFECV* module from *yellowbrick* library for selecting the optimum feature number. The Shapley additive explanations (SHAP) method is an innovative tool for explaining ML decision-making processes for datasets. The goal of the SHAP method is to present and explain the prediction with respect to the contribution of each feature to the predicted value. In RFECV, the features are ranked by a permutation importance measure. The SHAP algorithm was used for feature selection (Figure 2), as it provides more consistent and accurate importance values compared to the permutation approach. Ultimately, RFECV algorithms showed that 18 parameters are sufficient to explain nearly 90% of variances. Overall, 13 clinical and 5 laboratory variables were selected according to their SHAP values (Figure 2).

## Results

### Study Population Characteristics

A comparison of the demographic and clinical characteristics between the nonsevere and severe groups is presented in Table 3. Of the 437 patients, 304 (69.6%) met the primary outcome, requiring the escalation of care. Patients in the severe care group were significantly younger, with a median age of 23 months compared to 44 months in the nonsevere level of care group (*P*=.003). Additionally, the severe group had lower weight *z* scores (*P*=.045).

Key clinical differences included higher rates of respiratory distress (208/304, 82.9% vs 43/133, 17.1%; *P*<.001), abnormal lung sounds (277/304, 73.1% vs 102/133, 26.9%; *P*<.001), and hypoxia (240/304, 92.3% vs 20/133, 7.7%; *P*<.001) in the severe group. In terms of laboratory findings, the severe group had higher leukocyte counts (*P*=.005), neutrophil counts (*P*=.045), and lymphocyte counts (*P*=.001). Albumin levels were slightly lower in the severe group (*P*=.01). No significant differences were observed between the groups in gender distribution (*P*=.83), comorbidities (*P*=.26), recent antibiotic use (*P*=.17), or C-reactive protein levels (*P*=.76).

### Model Performances

In this section, we present a comparison of the performance of 16 different algorithms for raw and preprocessed datasets. We used various evaluation metrics such as accuracy, area under the receiver operator characteristic curve (AUC-ROC), recall, precision, *F*_1_-score, Cohen κ, and Matthews correlation coefficient to assess model performance. To analyze model performance, all prediction experiments were conducted using 10-fold cross-validation. Subsequently, the models were optimized, and their performances were evaluated on a balanced dataset using SMOTE-Tomek and feature selection. The performances of the three models with the highest performance (CatBoost, XGBoost, and LightGBM) were evaluated by applying hyperparameter optimization and ensemble methods. Table 4 compares the results obtained with CatBoost, XGBoost, and LightGBM among the optimized and nonoptimized results, as well as the results of the combinations with the highest performance from the basic ensembling methods (ensembling, blending, and stacking methods). The highest AUC-ROC value was achieved by using optimized LightGBM as the meta-model in the stacking method.

**Table 4.** Comparative performance of machine learning models for the escalation of care prediction. Italicized values represent the highest scores for each column.

Model	Accuracy	AUC-ROC^a^	AUC-PRC^b^	Recall	Precision	*F*_1_-score	Cohen κ	MCC^c^
CatBoost^d^	0.77	0.85	0.94	0.75	0.91	0.82	0.52	0.54
LightGBM^e^^,f^	0.80	0.87	0.96	0.79	0.92	0.85	0.58	0.59
XGBoost^f^^,g^	0.77	0.83	0.96	0.72	*0.94*	0.82	0.54	0.57
Ensembling^h^	0.77	0.86	0.95	0.72	*0.94*	0.82	0.54	0.57
Stacking^i^	0.80	*0*.88	0.96	0.79	0.92	0.85	0.58	0.59
Blending-1^j^	0.77	0.86	0.96	0.75	0.91	0.82	0.52	0.57
Blending-2^k^	*0*.85	0.84	0.96	*0*.95	0.85	*0.90*	*0.63*	*0.64*

a AUC-ROC: area under the receiver operating characteristic curve.

b AUC-PRC: area under the precision-recall curve.

c MCC: Matthews correlation coefficient.

d The performance of unoptimized CatBoost.

e LightGBM: light gradient boosting machine.

f The performance values obtained after optimization of XGBoost and LightGBM.

g XGBoost: extreme gradient boosting.

h The performance of the optimized LightGBM ensembling method, which achieved the highest results among CatBoost, XGBoost, and LightGBM algorithms.

i The performance of the model with optimized LightGBM as a meta-model in the stacking method, as it showed the highest performance.

j The combination of optimized LightGBM and XGBoost with higher performance in the blending method.

k Using the top-5, highest-ranked clinical features, the peak performance was realized by using a method that incorporated the optimized CatBoost, LightGBM, and XGBoost models.

In addition to the metrics reported in Table 4, we evaluated the performance of the *Blending-2* model using the precision-recall curve metric, which is particularly useful for imbalanced datasets. The precision-recall curve plot for this model, using the top-5 ranked clinical features, is provided in Multimedia Appendix 1. The model achieved a strong average precision-recall score of 0.96, further highlighting its robustness in handling imbalanced data.

### Feature Importance

The optimized LightGBM in the model, developed with balanced and feature-selected data, was responsible for the attainment of the highest performance. Upon evaluation of clinical features according to SHAP values, a ranking was established based on their feature importance scores, with the highest score being garnered by the top-5 clinical features (hypoxia, respiratory distress, age, *z* score of weight for age, and antibiotic usage before admission; Multimedia Appendix 2). The application of a workflow using these 5 features, as done previously, resulted in the highest accuracy performance (84%), which was achieved through the use of the ensemble method, incorporating the blending method of the optimized CatBoost, LightGBM, and XGBoost models.

## Discussion

Pneumonia, the leading cause of childhood mortality, is also one of the most common causes of hospitalization [3,22]. It remains a significant global health burden, particularly in children aged <5 years, where timely and accurate clinical management is crucial for reducing mortality [8]. While prevention strategies are well documented, the clinical challenge lies in efficiently identifying patients who require escalated care. In this study, we present a contemporary approach to building an ML-based, prognostic care referral decision support tool that assists primary care physicians in determining where the case should be managed with an accuracy of more than 80%.

Today, there is widespread knowledge of the prevention, diagnosis, treatment, and management of complications in CAP, but due to resource limitations, it is not possible for all physicians and patients to benefit from this [14]. Recent advancements in medical informatics have the potential to reduce health care disparities and empower physicians in resource-limited settings [11-15], offering new hope for identifying high-risk populations and preventing mortality where current methods fall short.

The recent COVID-19 pandemic has impacted several medical fields, including the disruption of research practices by shifting researchers’ focus and patient recruitment [23,24] and significantly reducing the incidence of non–COVID-19 pneumonia by preventing transmission [25-27]. In the current postpandemic state, non–COVID-19 childhood pneumonia remains a global health concern, especially in resource-limited settings according to the most recent reports [2], with respiratory infections likely to rise again as pandemic measures have already been eased [28]. Now, focusing back to reducing the mortality of CAP is critical to ensure pediatric pneumonia care benefits from recent advancements that COVID-19 provided [29,30]. This study, built primarily on prepandemic cases, provides a foundational context for future studies on CAP using ML in the postpandemic era.

Since March 2020, a substantial amount of data about COVID-19 have been published, including COVID-19–related artificial intelligence studies focused on pneumonia diagnosis by radiological findings [31]. However, pneumonia diagnosis is clinical, and routine chest radiographs are not necessary for the confirmation diagnosis [32] and do not improve outcomes [33]. In addition, chest radiography can be used only in inpatient settings to identify complications or evaluate response to treatment.

Although strong diagnostic support algorithms have been published in pneumonia-related studies in recent years, there is still a need for prognostic studies for pneumonia management [31]. Determining the severity of a disease or predicting its prognosis answers essential questions of physicians in medical decision-making, such as “Where should it be treated? Outpatient? ICU?” “Which therapy should I start? How long should I give it?” and “When should I discharge the patient? When should I call for control?” There are several studies and guidelines in the literature for severity assessment and prognosis prediction of pneumonia [9,10,34]. For the majority, mortality and the development of complications were the primary outcomes, and clinical, radiological, and laboratory variables are the key predictors. Yet, there is a limited number of studies predicting required referral to tertiary care based on basic clinical and laboratory features available in primary care settings [15].

This study reviewed important pneumonia prognostic predictors of children hospitalized in a major academic medical center. The primary outcome of interest was the level of care severity, classified as severe or nonsevere based on the need for pediatric intensive care unit admission or oxygen/ventilation support. The main objective of this study was not only to build the best model but also to answer the primary care physician’s question: “Where should the case be managed?” Our model demonstrated promising predictive accuracy, with an AUC-ROC exceeding 0.85 and an accuracy of 77% to 88% (Table 4). The key clinical features identified—hypoxia, respiratory distress, age, *z* score of weight for age, and complaint period (Multimedia Appendix 2)—align with existing clinical guidelines, which emphasize the importance of respiratory and nutritional status in predicting disease severity [33-36].

In this study, we used SMOTE-Tomek, a method proven effective in medical tasks, to address class imbalance without losing valuable clinical information [37,38], which was essential given the significantly imbalanced and small sample–sized dataset. Additionally, we used RFECV and SHAP, both of which have been established as robust methods in previous studies [11,39,40], for feature selection. These techniques not only improved our model’s performance but also allowed us to isolate the most clinically significant features (Figure 2, also see Multimedia Appendix 2), enabling clinicians to decide using their own skills without involving additional diagnostic tools.

The clinical application of a prognostic care decision model is particularly relevant in settings where early and accurate escalation of care is needed. For example, by focusing on these top-5 clinical features or using a decision support tool like ours, even less experienced primary care physicians could assess risk and anticipate tertiary care referrals without advanced diagnostics. Additionally, in emergency settings, these tools could assist in triaging patients to prioritize those needing immediate respiratory support or mechanical ventilation, allowing earlier interventions and more effective resource allocation—crucial for LMICs—potentially reducing morbidity and mortality.

One significant limitation of this study is its reliance on data from a single tertiary hospital (Hacettepe University), which may limit generalizability. While the dataset includes patients referred from both urban and rural areas, the focus on a tertiary center introduces a selection bias, as most cases represent severe care levels (304/437, 69.6%). This is likely because less severe CAP cases are managed in primary or secondary care, not referred to tertiary centers, limiting the model’s applicability in less severe cases. Additionally, the relatively small sample size of 437 patients limits the model’s generalizability, as larger datasets are typically needed to optimize ML models and ensure robust performance across diverse populations. Expanding the dataset to include patients from multiple centers, especially primary and secondary care institutions, could improve the model’s generalizability and applicability. Lastly, the retrospective nature of the data and the missing time frames of tertiary care unit transfers may not fully capture real-time clinical decision-making or the urgency of care decisions.

In conclusion, this study demonstrates the feasibility of developing an ML-based prognostic decision support tool for childhood pneumonia referral, with an accuracy of 77% to 88%. Incorporating foundational clinical skills for key prognostic predictors with advanced data science methods holds promise for improving pneumonia outcomes by accurately predicting the need for the escalation of care.

## Supplementary material

10.2196/57719 Multimedia Appendix 1 Precision-recall curve (PRC) for the blending model with top 5 features.

10.2196/57719 Multimedia Appendix 2 Shapley additive explanations (SHAP) values forward selection method.

## Abbreviations

AUC-ROC: area under the receiver operator characteristic curve
CAP: community-acquired pneumonia
IMCI: Integrated Management of Childhood Illness
LightGBM: light gradient boosting machine
LMIC: low- and middle-income country
ML: machine learning
RFECV: recursive feature elimination with cross-validation
SHAP: Shapley additive explanations
SMOTE: Synthetic Minority Oversampling Technique
WHO: World Health Organization
XGBoost: extreme gradient boosting
