# Supplementary figures and images for: Predicting Escalation of Care for Childhood Pneumonia Using Machine Learning: Retrospective Analysis and Model Development
Source: JMIRx Med. 2025 Mar 4;6:e57719. doi: 10.2196/57719 (PMC11896559; doi:10.2196/57719)

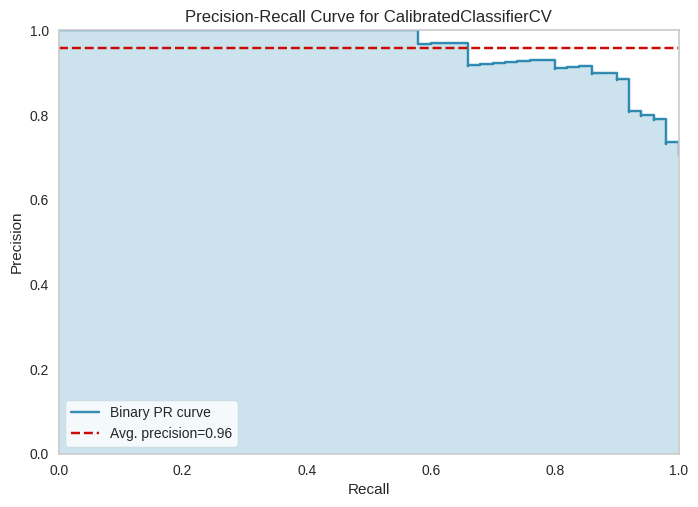

Supplement: Multimedia Appendix 1 [file xmed-v6-e57719-s001.png]

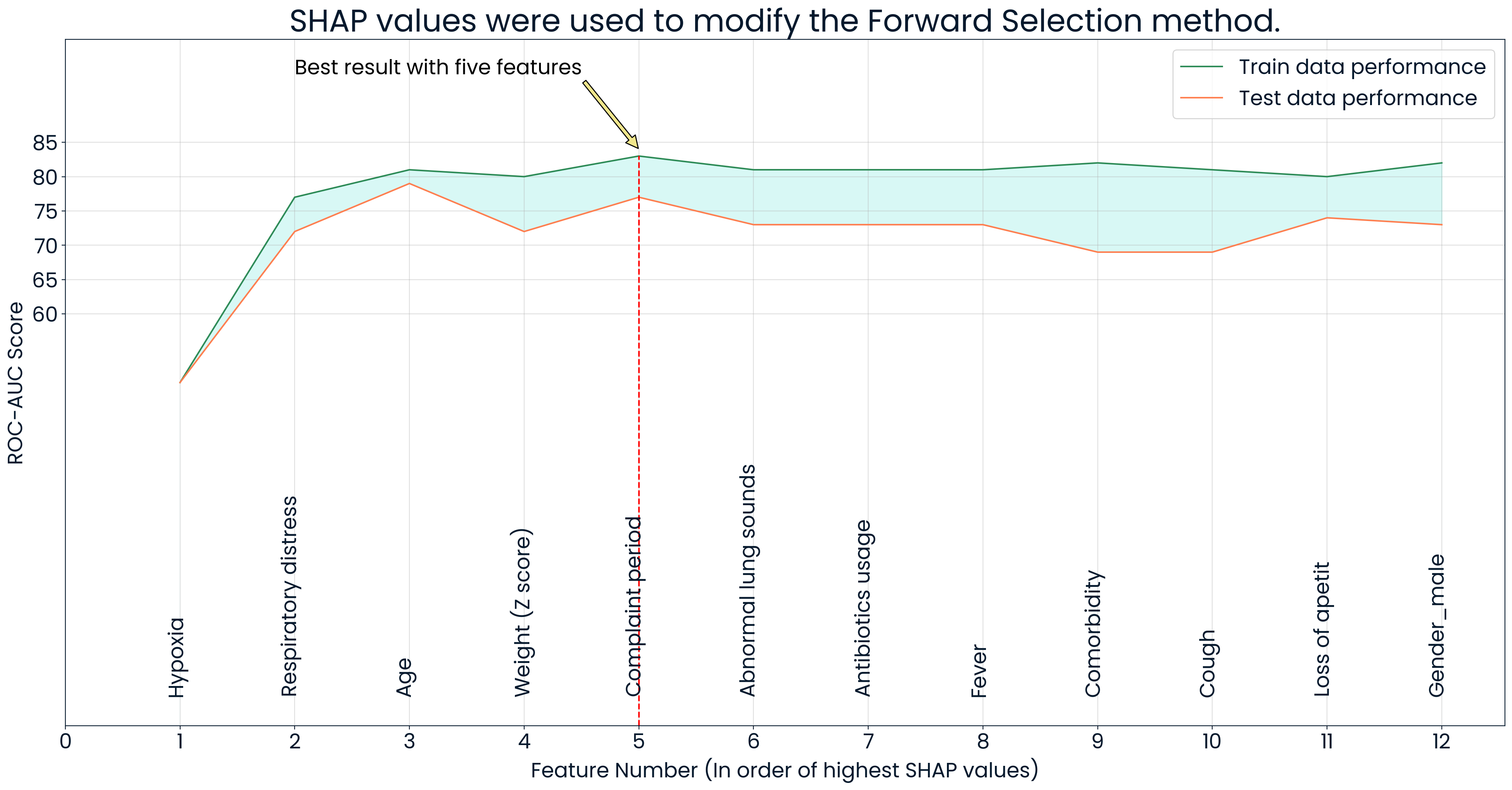

Supplement: Multimedia Appendix 2 [file xmed-v6-e57719-s002.png]
